# The Communities Organizing for Power Through Empathy (COPE) Community-Based Intervention to Improve Adult Mental Health During Disasters and Crises: Protocol for a Stepped-Wedge Cluster Randomized Trial

**DOI:** 10.2196/63723

**Published:** 2025-05-20

**Authors:** Jennifer Scott, Tara Powell, Natasha M Lee-Johnson

**Affiliations:** 1 School of Social Work Louisiana State University Baton Rouge, LA United States; 2 School of Social Work University of Illinois Urbana-Champaign Champaign, IL United States

**Keywords:** mental health, community-based group mental health intervention, disaster, community-based participatory research, stepped wedge cluster randomized trial, Gulf Coast

## Abstract

**Background:**

Severe weather events, exacerbated by climate change, can lead to hardships such as displacement, resource scarcity, and social network disruptions. Such disasters impact mental health, triggering conditions such as anxiety, depression, and posttraumatic stress disorder. For communities in the Gulf South, the increasing frequency of disasters often further exacerbates already disastrous levels of inequality. In this context, there is an urgent need for evidence-based, multilevel, community-based interventions to support individual and community mental health and resilience.

**Objective:**

This protocol describes the design of a community-based participatory research (CBPR) study to improve individual and community mental health in Gulf South communities by examining the implementation and effects of the multilevel, community-based intervention, Communities Organizing for Power through Empathy (COPE). Specifically, this study aims to (1) examine factors affecting the implementation, effectiveness, and adoption of the COPE intervention and (2) test its effects on mental health and community resilience. We hypothesize that participants in the COPE intervention will experience greater reductions in psychological distress (eg, perceived stress, anxiety, and depression); improvements in protective factors (eg, social support and coping self-efficacy); and community participation as compared with an attention control group.

**Methods:**

The Consolidated Framework for Implementation Research guides our analysis of data collected from surveys, fidelity and field notes, interviews, and focus groups to examine aim 1. We examine aim 2 primarily via a 2-arm pragmatic stepped-wedge cluster randomized trial (SW-CRT) with individuals (approximately n=300) in clusters (faith-based and secular community-based organizations; approximately n=15) in a disaster-prone community in the US Gulf Coast. The cluster-based design implemented in steps supports the community-based nature of the study where timelines differ by organization. A total of 5 self-assessments will be conducted both in person and via email at later time points. We will integrate mixed methods in our analyses for aim 1 by combining themes from interviews and focus groups with implementation measures, and for aim 2 by constructing a data matrix to combine findings from the SW-CRT with thematic analyses of field notes and interviews.

**Results:**

The SW-CRT is being conducted from June 2022 to June 2025. Recruitment began in April 2023, to conclude in spring 2025, to assess mental health, social support, and community resilience at 5 time points. Data analysis and dissemination of results are expected by spring of 2026.

**Conclusions:**

This protocol is among the first to use a CBPR approach to examine the implementation and effectiveness of a multilevel intervention on psychological distress and resilience. This study provides new insights into how CBPR can enhance intervention implementation research and expand the evidence on community-based mental health interventions during disasters. Policy makers should consider integrating CBPR approaches into disaster response frameworks to ensure culturally relevant and sustainable outcomes.

**Trial Registration:**

ClinicalTrials.gov NCT06093737; https://clinicaltrials.gov/study/NCT06093737

**International Registered Report Identifier (IRRID):**

DERR1-10.2196/63723

## Introduction

### Background

Exposure to severe weather events can have far reaching consequences as affected individuals and communities confront hardships such as displacement, property damage, and resource scarcity [1]. These catastrophic events also often disrupt social networks and community structures, further complicating recovery [2,3]. Climate change, linked to greater frequency of these severe weather events and environmental disasters, such as hurricanes and tropical storms, has increased the threat to communities across the globe [4]. For many communities, these events exacerbate already precarious living situations and accompanying high levels of psychological distress [5-7], often persistent across generations, as evidenced by higher levels of posttraumatic stress symptoms (PTSS) found among children [8]. In addition, individuals and communities have become so disconnected that the US surgeon general advisory in 2023 stated that the country was facing an “epidemic” of social isolation [9]. In this context of increasing risk, there is an urgent need for evidence-based multilevel interventions that support individual and community mental health. This protocol describes a study that aims to add to that evidence base, detailing a community-based participatory research (CBPR) approach to examining the implementation and effectiveness of the multilevel, community-based intervention, Communities Organizing for Power through Empathy (COPE) [10].

Designed to address the compounded psychological stresses that communities face during crises, COPE expands resources available to support communities during crises and disasters by integrating interventive and community-building components with training on providing support using elements of psychological first aid (PFA) and mental health first aid (MHFA) [11-13]. The COPE intervention is particularly important for the Gulf Coast region, where the enduring legacies of slavery and systemic racism have contributed to disastrous levels of inequality [14] and the impacts of climate change and frequency of disasters further exacerbate disparities [15,16]. CBPR is a collaborative approach to research that establishes structures to meaningfully engage affected communities in partnership with researchers in all aspects of the process [17-20]. The CBPR project that developed the COPE intervention is now in the implementation phase and aims to address this heightened need for mental health support in a context of limited access to services. Adapted from an intervention that has shown sustained reductions in PTSS for participant survivors of hurricanes Harvey and Maria [21], the COPE intervention uses group process, psychoeducation, and dynamic activities to strengthen individual and collective coping strategies, as well as individual and community relationships.

### Mental Health and Disasters

The mental health impact of environmental disasters is both profound and multifaceted [22,23]. Trauma and loss associated with disaster exposure can trigger a range of conditions, including anxiety, depression, and posttraumatic stress disorder (PTSD) [5,6]. Rates as high as 59% for clinical depression have been found among survivors after severe flooding in China, and as 70.5% for PTSD among survivors after Hurricane Sandy in New York [5]. Furthermore, the psychological effects experienced from a disaster can persist, seen in higher rates of mental health disorders observed years following the disaster [24]. A study conducted 3 years after Hurricane Katrina devastated the Gulf Coast and the city of New Orleans in the United States found that 33% of survivors reported probable PTSD symptoms [25]. Another conducted 12 years after Katrina found that 1 in 6 low-income survivors still reported symptoms of PTSD, and the rates of psychological distress remained higher than before the hurricane [26].

The adverse effects of a disaster are shown to be further exacerbated when individuals and communities already face deficits or inequalities in resources or supportive services, and particularly when those inequities have spanned generations [14,27-30]. Low-income families, older adults, people with disability, immigrants, children, those who are imprisoned [31,32], and women [33] are disproportionately impacted, often due to limited resources and infrastructure with which to effectively prepare for, respond to, and recover from disasters [28,34]. Limited financial or social resources, coupled with reduced access to basic health and mental health services, can impede capacity to rebound from such events [35]. This can lead to enduring psychological distress, deepening socioeconomic disparities, and a prolonged disruption of community cohesion and recovery [31,34,36].

The development of the COPE intervention and this study were motivated by our awareness that many of the factors associated with increased risk of experiencing adverse psychological outcomes after a disaster, such as prior trauma [37], limited income [27], and low levels of social and community support [38], are omnipresent in our community and the Gulf South. In addition, uncertainty and chronic stress associated with disaster exposure, frequent in the region, have been shown to exacerbate existing mental health conditions [39,40]. Studies have shown, however, that protective factors, including individual adaptive coping strategies [41-43], self-efficacy [44-46], and spirituality [47-50] interpersonal social support and relationships, and community or neighborhood cohesion [33,51], can reduce the likelihood of experiencing adverse mental health outcomes after a disaster. This study aims to examine whether the COPE multilevel intervention, designed to enhance individual protective factors (eg, adaptive coping), and delivered in a format designed to strengthen social capital (ie, interpersonal and community protective factors such as social support, relationships, and community cohesion), could improve individual mental health outcomes in communities with persistent high levels of inequity and disaster-related stress.

### Disaster-Related Mental Health Support Interventions

Mental health support services are important in all phases of a disaster (preparedness, response, and recovery) to help individuals and communities cope with trauma, prevent long-term psychological consequences, and support healthy recovery. In the preparedness phase, disaster mental health programs are designed to psychologically prepare individuals and communities for potentially traumatic events. Education and training interventions in this phase enhance the community’s capacity to respond to mental health needs if a disaster occurs [52].

One widely disseminated preparedness training, PFA, is designed to increase the capacity of non–mental health specialists to provide basic safety and stability to survivors during the response phase [11,53]. Core elements of PFA include addressing basic needs, mitigating harm, providing reassurance and safety, and connecting survivors to supports and services [11,54]. A similar training intervention, MHFA, is designed to equip nonspecialists with the knowledge and skills to identify and respond to individuals experiencing psychological distress [55]. Specifically, MHFA focuses on identifying signs of mental illness and substance abuse, providing basic psychological support, and connecting distressed individuals to professional support services [55]. Both PFA and MHFA primarily use didactic lectures and instruction focused on disseminating information instead of delivery methods that engage participants in group processing, interactive learning through hands-on activities, and reflection. Although MHFA and PFA have been shown to increase participant knowledge on providing mental health support to disaster survivors, research on how the knowledge and skills gained is deployed during disasters is limited [13,54].

During the disaster response phase, mental health services focus on the immediate needs of survivors [34,56]. Disaster mental health response efforts generally involve identifying and triaging individuals to crisis counseling services or providing immediate stabilization to distressed survivors [56]. However, crisis counseling varies widely in approach and method, making it difficult to standardize care and measure outcomes [56]. Psychological debriefing (PD), a brief intervention delivered by a mental health professional as a single individual or group session within 72 hours of a traumatic event, includes discussing, validating, and normalizing survivors’ trauma response [56]. There is mixed evidence on the effectiveness of PD: some studies suggest debriefing can reduce PTSS [57] and others have indicated PD may cause further distress [58].

The recovery phase focuses on the long-term mental health and well-being of survivors. It also contributes to preparedness and mitigation. Formal mental health treatment is one category of intervention provided to support survivors exhibiting clinical manifestations of distress [59]. Cognitive behavioral therapy, narrative exposure therapy, and other forms of psychotherapy have all been found to reduce clinical rates of PTSD, depression, and anxiety in disaster survivors [60,61]. Other mental health supports provided during the recovery phase include community-based wellness and resilience-building psychosocial interventions, generally designed for specific groups (eg, teachers, social service, or health care providers). One brief intervention, Caregivers Journey of Hope (CJoH), is designed to increase coping and reduce stress among disaster-affected social service providers [62]. It has been found to significantly reduce perceived stress and improve social support and knowledge of community resources [62]. Another intervention, Resilience and Coping for the Healthcare Community (RCHC), incorporates psychoeducation, action learning, and solution-focused strategies to reduce distress and increase social and peer support among health care providers [21,62,63]. A study assessing the effects of the RCHC among health care employees after hurricanes Harvey and Maria in 2017 found a significant likelihood of experiencing reductions in PTSS, anxiety, and secondary traumatic stress at the *P*<.01, *P*<.05, and *P*<.05 levels, respectively, at week 12 for those who received the RCHC as compared with a control condition [21]. Targeting both the recovery and preparedness phases, the COPE intervention being examined with this protocol adapted the RCHC for delivery to laypersons, incorporating illustrated materials to deepen conceptual understanding and foster dialogue across diverse literacy levels, content on historical trauma to acknowledge systemic factors, and sessions on supporting people in distress and identifying neighborhood resources to improve community response capacity [10].

Research examining the efficacy of disaster mental health interventions has primarily focused on the effectiveness of interventions designed to treat clinical diagnoses such as PTSD, anxiety, and depression [34,64], or on trainings that prepare individuals to provide basic psychological support [34,65]. Emerging evidence suggests community-based interventions, such as the CJoH and RCHC, improve individual mental health after the disaster [21,62]; however, more research is needed on their applicability across contexts and with diverse populations, as well as on how they affect individual mental health and community resilience [34,52]. In addition, as highlighted by Gil-Rivas and Kilmer [18], scholars have recommended “a CBPR approach to disaster preparedness and response that involves community-level organizing” as an effective strategy to strengthen community resilience in a culturally relevant and contextually appropriate fashion [66-68]. Better understanding the effectiveness and implementation of multilevel interventions designed for broad audiences that address mental health and well-being at the individual, interpersonal, and community levels is key to improving access during recovery [69].

### Rationale for the Study

The compounding psychological effects of successive disasters on the Louisiana Gulf Coast, including 5 named storms in the 2020 hurricane season followed by the catastrophic Hurricane Ida in 2021, in the context of the long-term stress of the global COVID-19 pandemic, brought to the foreground the need to consider mental health as a component of community resilience. Community resilience refers to a community’s capacity to prepare for, respond to, and recover from crises and disasters [70]. Important aspects of community resilience include strong community networks, local knowledge and response capabilities, and access to physical and mental health care [70].

Often, and particularly in the Gulf Coast region, it is neighbors and local community members who are the first to respond to a disaster [71]. This role that neighbors and local community members play in disaster response underscores the need for multilevel interventions to increase individual and community capacity to prepare for and cope in the aftermath of disasters. It also underscores the value of engaging community members in a CBPR approach to adapting and implementing those interventions. In response, the COPE curriculum [10] was developed via a CBPR project undertaken by the community organization Together Baton Rouge (TBR) and academic researchers. TBR is a 501(c)3 nonprofit and a broad-based organization [72,73], meaning that it is comprised of, and financially sustained by, other faith-based and secular organizations (community-based organizations [CBOs]) in the community that pay membership dues to support its work. This partnership structure thus provides a unique opportunity to test a distinct implementation design by delivering COPE at a variety of CBOs that are interconnected as members of the single committed partner organization, TBR.

This study responds to 2 primary questions about the COPE intervention model: (1) How do contextual factors affect intervention implementation? and (2) How does COPE affect individual, interpersonal, and community level well-being? We examined these questions using a pragmatic, stepped-wedge cluster randomized trial (SW-CRT) design with rolling enrollment. This design permits study of intervention effects in a context where individual randomization was not feasible given the group-based intervention and its delivery to individuals associated with the cluster (community institution) where they participate. In addition, the stepped roll out better supported the community-based nature of the study in which all clusters were not ready to deliver at the same time, and all expected to participate in the intervention.

### Aims and Hypotheses

**Figure 1:**
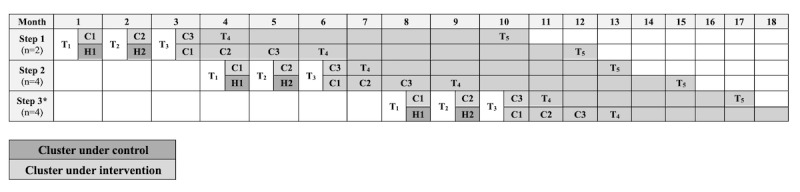
Timeline for Communities Organizing for Power through Empathy (COPE) stepped-wedge cluster randomized trial design and assessment. C1-3: COPE sessions 1-3; H1-2: house meetings 1-2; T1: baseline measurement period; T2-5: time points 2-5. *Steps 4 and 5 follow the same sequence as steps 1-3.

In this paper, we describe the design of our mixed methods study that includes a pragmatic 2-arm SW-CRT [74] that examines the effectiveness and implementation of COPE, a group psychoeducational intervention designed to support individual and community mental health. The SW-CRT research design randomly assigns clusters (eg, communities and organizations) to transition from control to intervention in phases, until each eventually receives the intervention [74]. This research design supports phased community engagement and recruitment by sequentially introducing interventions, helping to reduce staff and organization burden. The aims of this SW-CRT are 2-fold. First, we aim to examine contextual factors, such as organization type and timing of delivery, to understand how they affect implementation, efficacy, and adoption of the COPE intervention. We hypothesize that cluster-level factors—such as organizational support, participant engagement, and facilitator training—and individual-level factors—such as age and education level—will influence feasibility, implementation, and acceptability of the COPE intervention. In addition, we hypothesize that the interrelationship between clusters as part of a larger organization will improve recruitment both at the cluster and individual level. Second, we aim to test the effectiveness of the COPE intervention [10], compared with an attention control arm (house meetings), in reducing mental health distress and strengthening coping, social support, and community engagement across 5 time points (Figure 1). We hypothesize that individuals who participate in the COPE intervention will report greater reductions in psychological distress symptoms (perceived stress, depression, and anxiety) than the attention control group, alongside significant increases in protective factors (coping self-efficacy, social support, and community resilience).

## Methods

### Overview and Study Design

This study uses a pragmatic mixed methods approach to examining the effectiveness and practical relevance of COPE in a real-world context [75]. We will collect and examine qualitative data to address aim 1, capturing contextual information about cluster-level and individual-level factors that influence the feasibility, implementation, and acceptability of COPE. We will approach aim 2, the test of intervention effectiveness, using a SW-CRT with a rolling enrollment design. As a pragmatic 2-arm SW-CRT, the study has two conditions or arms: (1) the intervention arm, in which clusters of participants begin by engaging in the COPE intervention, and (2) the attention control arm, in which clusters of participants begin by attending “house meetings” (listening sessions) before switching to the COPE intervention. A cluster is defined as a single CBO (or in a rare case, a pair of related CBOs). Clusters are eligible to participate if they are CBO members of TBR. Steps are clusters grouped by availability (ie, feasible start date as provided by CBO leadership). At the initiation of a step and before participant enrollment, clusters are randomized into study arms.

As a SW-CRT, every cluster will provide before and after observations, and will switch from control to intervention, but not at the same point in time (Figure 1) [74]. At study outset, clusters are assigned by the first author to 1 of 5 sequential steps: 2 in step 1, 4 in steps 2 and 3, and 6 in steps 4 and 5 (Figure 1). At the initiation of a step, clusters will be randomized by the first author into the intervention and control arms using a random number generator. Allocation into intervention or control arms will not be concealed at either the cluster or individual level, nor will clusters or participants be blinded, as doing so is not feasible given the group-based nature of the intervention and community-based nature of the study implementation. We will include controls to account for temporal and enrollment differences. Presurvey data collection will occur immediately before the start of the intervention within each cluster. After the third data collection time point, the clusters in the attention control arm will switch to receive the COPE intervention (see Figure 1 for the design timeline).

To accomplish aim 1, we are using the Consolidated Framework for Implementation Research [76] to examine factors that affect intervention implementation and program efficacy, as well as standardized measures of perceptions of feasibility, acceptability, and cultural appropriateness. We will conduct quantitative assessments, interviews, and focus groups with community facilitators (CFs), institutional leaders, and participants (see Multimedia Appendix 1 for facilitator interview guide). In addition, to align with our CBPR approach, our research team includes a group of community members we refer to as the “CBPR strategy team” who guided the adaptation process that developed COPE and will guide the study design, implementation, and analysis of findings. For example, we presented them with options for measures of mental health and discussed the overall survey design. The team chose measures and approved the final survey instrument. We also worked with the team to discuss options for implementation of the COPE sessions and recruitment. As we analyze data, we will review findings and papers developed with the team, along with other community members. In our analyses for aim 1, we will integrate the methods by combining themes from interviews and focus groups with analyses of implementation measures.

To accomplish aim 2, we are collecting cohort data from participants in clusters in both arms assessed along measures of mental health, coping, social support, and community resilience at 5 time points (see Figure 1 for study design and data collection time points). Participants will complete assessments at each time point before content delivery using Qualtrics survey software loaded on a tablet. In cases where participants are unable to use a tablet (eg, discomfort with technology or visual impairment), they will complete the assessment on paper and research staff will enter the data into the software. Participants will be given a US $10 gift card at the completion of each questionnaire.

### Ethical Considerations

**Figure 2:**
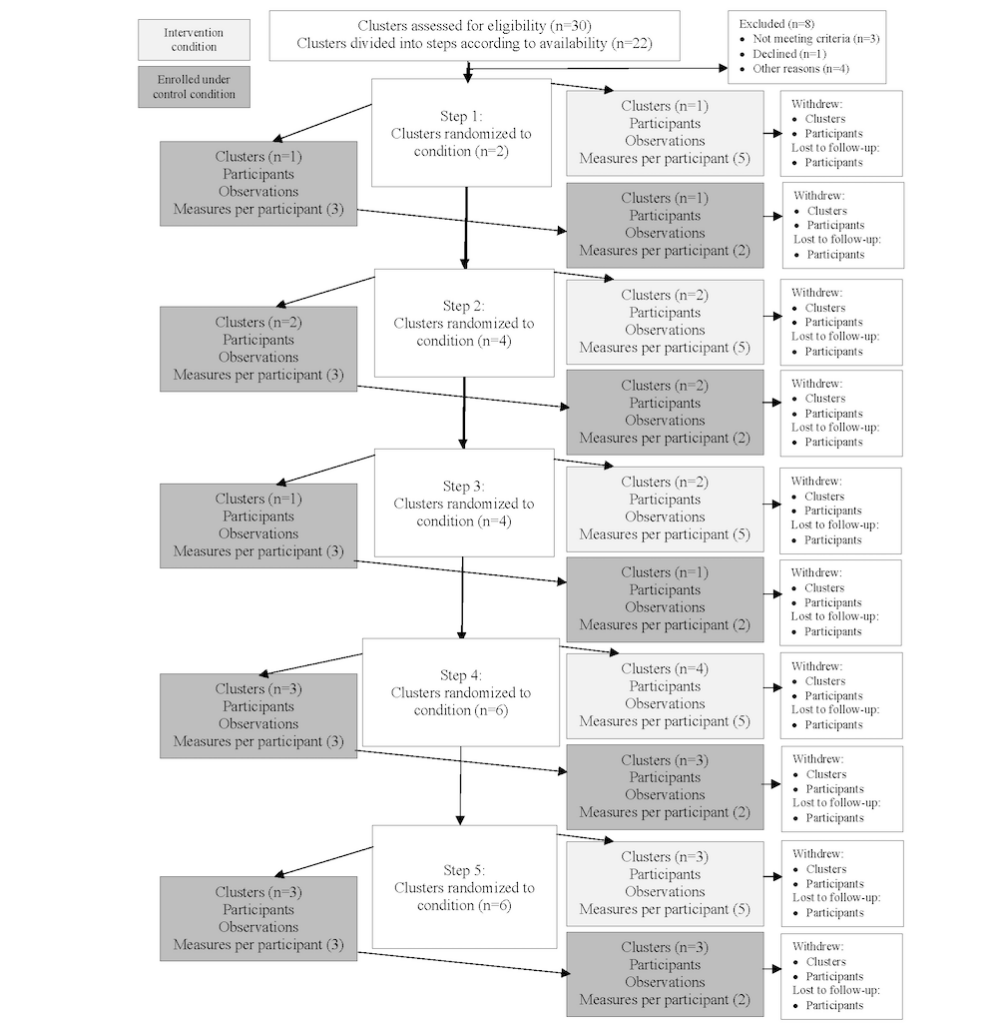
Communities Organizing for Power through Empathy (COPE) stepped-wedge cluster randomized trial enrollment flow diagram.

The current SW-CRT is approved by the institutional review board of Louisiana State University (IRBAM-21-1391) and funded by the Gulf Research Program of the National Academies of Sciences, Engineering and Medicine. All enrollees will provide informed consent. The trial has been registered on ClinicalTrials.gov (NCT06093737) and is following the SW-CRT extension for the CONSORT-SPI (Consolidated Standards of Reporting Trials–Social and Psychological Intervention) guidelines for reporting (see Figure 2 for the flow diagram) [77-79].

Research staff and coinvestigators will undergo human subjects training by completing the Collaborative Institute Training Initiative social and behavioral health training module. Individual data are confidential; however, as a community group-based intervention, cluster- and individual-level participation itself is not. To account for differential exposure times between clusters inherent to a SW-CRT design, we will clearly communicate timelines to manage expectations and provide adequate referrals to mental health resources.

To ensure data confidentiality, participants’ responses will be self-reported, and a unique identifier will be assigned to link surveys from the same participant across time. With regard to the qualitative data, at the start of each session of the COPE intervention, participants will discuss confidentiality guidelines, emphasizing respect for privacy to minimize risks of unintentional disclosures. Similarly, at the start of each focus group and interview, confidentiality will be discussed as part of the informed consent process. After transcription of qualitative data, names will be replaced with pseudonyms.

Only the researchers listed on the institutional review board approval will explain, collect, and manage informed consent. Before initial data collection, researchers will explain the study’s purpose, procedures, potential risks, benefits, and right to refuse, as well as the limits of confidentiality, using a plain language script developed by the authors with feedback from the strategy team. In addition, we will allow time for question and answer to ensure participants understand the information before consenting. Researchers will collect signed consent forms that are stored in a locked filing cabinet separate from the data. A reminder of their rights as research participants will be provided before each subsequent assessment, even though signatures are only collected at the initial assessment. Enrollees of both trial arms will be compensated for participation with a US $10 gift card provided for each survey, to a potential total of US $50, and food provided at each in-person or data collection session. CFs will also sign their informed consent to participate in their initial training, be compensated for their time in training and facilitation at a rate of US $600 per series plus US $400 for training (1-time payment, approximately US $50 per hour), and be provided all materials required for facilitation.

### Sampling Strategies and Sample Size

Multilevel mixed methods, with convenience, purposive, and probability sampling techniques at different levels of analysis, in this case clusters and individuals, will be used as different strategies are required for each aim. Convenience sampling will be applied in the initial phases of the study during the adaptation process to pilot-test the COPE intervention’s components with accessible participants from across the clusters to ensure broad acceptability and commitment to participation in the SW-CRT. For aim 1, maximum variation sampling will be used to gain perspectives from a heterogenous pool of participants and facilitators [80]. This will include purposive sampling of participants from different institutions to gain a diverse set of perspectives on barriers or facilitators that impact implementation of COPE.

For the SW-CRT component of aim 2, we did a power analysis to estimate the required sample size for a target power of 80%. We assumed a standardized moderate effect size of 0.3, 5 time points, a conservative intraclass correlation coefficient (ICC) of 10% (10% site-level and 90% person-level variation), and a within-person correlation of 0.3. The power analysis results indicated that 300 participants at 15 sites with 20 participants per site achieves the target power. We also did sensitivity analyses varying the effect size, ICC, within-person correlation, and number of sites, and with very conservative estimates the target power can be achieved.

After clusters are randomized into groups, participants will be recruited by CFs and project staff using convenience sampling techniques. As one cluster includes a large Spanish-speaking population among its membership, COPE will be delivered to participants in that cluster in Spanish by native Spanish-speaking bilingual facilitators and participants will be provided materials in Spanish.

### Study Setting, Participants, and Eligibility Setting

Louisiana, overall, is estimated to be affected with higher rates of mental health conditions than the national population. As of February 2021, about 715,000 adults in Louisiana had a mental health condition [81]. Nearly half (47.5%) had reported symptoms of anxiety or depression and, that same year, 720 lives were lost to suicide [81]. Despite this need, approximately 3.4 million people in Louisiana lived in a community without sufficient mental health providers [81]. In 2022, about 16% of adults in East Baton Rouge Parish reported experiencing frequent mental health distress [82].

The trial is being conducted at TBR and the cluster CBO locations. The inclusion and exclusion criteria for trial participants are listed in Textbox 1.

**Eligibility criteria.**

**Inclusion criteria**

- Currently aged ≥18 years
- Staff, members, or affiliates of a community-based organization (CBO) affiliated with Together Baton Rouge (TBR)
- Willing to participate in the intervention

**Exclusion criteria**

- Not aged ≥18 years
- Not staff, members, or affiliates of a CBO affiliated with TBR
- Unwilling to participate in the intervention

### Recruitment and Enrollment

CBO clusters are members of TBR selected to account for geographic and socioeconomic diversity. Clusters are enrolled by program staff who work with the CBO leadership to identify qualified CFs and enroll eligible participants. Engagement efforts emphasize building relationships with trusted local leaders and inclusive outreach that uses culturally relevant materials and ensures transparency. CFs deliver the intervention and recruit participants. Licensed mental health professionals (eg, licensed master or clinical social worker, licensed professional counselor, or licensed marriage and family therapist) or individuals with experience in group or community facilitation, who are affiliated with a TBR institution, and who are willing to commit to the training and delivery of all components of the intervention are eligible to be a CF. To every extent possible, CFs are members or strongly affiliated with the cluster where they are delivering the COPE intervention. In some cases, however, trained CFs may facilitate for a cluster with which they are not strongly affiliated.

Potentially eligible participants will be recruited by the CFs and leaders at each participating cluster CBO. This will be accomplished by a combination of posting fliers, announcements at critical meetings, word of mouth, phone, and social media, as appropriate to each cluster. Enrolled participants will be those who signed the informed consent and completed the baseline (T1) assessment.

### Intervention Arm: COPE

COPE is a 3-session group psychoeducational workshop series we developed that combines psychoeducation and peer support strategies to effect individual, interpersonal, community, and ultimately, societal change. The primary COPE session (C1) is adapted from the RCHC [63] curriculum developed by the second author (TP) and colleagues to provide psychosocial support to professional caregivers, specifically health care and social service providers. During the COPE adaptation, we engaged a team of mental health professionals from diverse backgrounds (in terms of race and ethnicity, gender, national origin, age, and primary language) in a collaborative process to tailor the RCHC to fit the unique needs of Gulf Coast communities at risk of or having survived disasters. Key elements of the adaptation include illustrated materials to engage varied literacy levels and language backgrounds, addition of content on historical and systemic trauma, and incorporation of activities on how to support individuals in distress and build community support.

**Figure 3:**
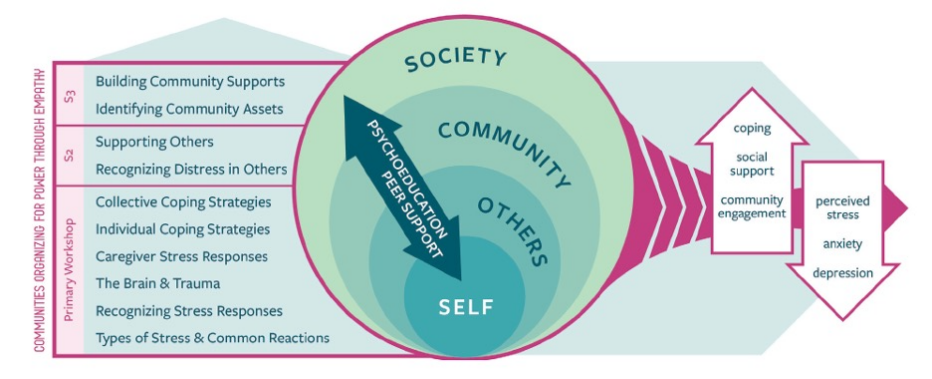
Communities Organizing for Power through Empathy (COPE) conceptual model. S2: COPE session 2; S3: COPE session 3.

As illustrated in the COPE conceptual model in Figure 3, the 3 sessions are designed to increase well-being at the individual (coping capacity), interpersonal (social support), and community (engagement) level and decrease adverse individual-level mental health outcomes (perceived stress, anxiety, and depression). Designed to normalize common reactions to stress after traumatic events, the psychoeducational component includes information on stress reactions and their biophysical components (eg, neurological trauma response), reactions common to caregivers, and adaptive coping strategies. The curriculum is grounded in active learning principles, engaging participants in Socratic dialogues about concepts and practical activities. In addition, COPE delivery integrates a group practice model that allows for within-group dialogue, social learning, and cooperation to support group reinforcement of positive change [83]. Through group process incorporated with the discussion of psychoeducational components, COPE helps to reduce isolation by creating an environment in which participants share their experiences and support each other, thus strengthening relationships and social networks.

**Figure 4:**
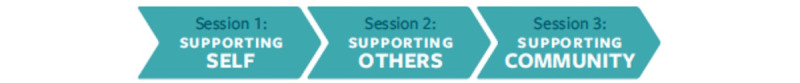
Communities Organizing for Power through Empathy (COPE) session topics.

As shown in Figure 4, the COPE series consists of the primary session 1 (C1, 4 h), focused on recognizing stress in oneself and developing healthy coping strategies while simultaneously building relationships to increase social and community support, and 2 additional follow-up sessions (C2 and C3, 90 min each). Each workshop session engages participants in group exercises that provide information in stress and trauma, skills training on recognizing and supporting individuals exhibiting distress, identifying and developing healthy coping strategies, and identifying and building community-based support.

The second session (C2) focuses on supporting individuals in distress and the third (C3) focuses on building community support. More specifically, session 2 (C2) centers skill building for nonspecialists to increase their capacity to provide basic psychological support to individuals in distress, and it incorporates an interactive role play to provide an opportunity to practice skills learned. Session 3 (C3) centers community resilience, engaging participants in an interactive discussion to name community assets and then map those available within the geographic area of their institution. COPE has been standardized in a single manualized curriculum with accompanying large-format illustrated posters laminated for interactive use and participant workbook [10]. The COPE participant workbook has been translated into Spanish and validated using back translation. For the SW-CRT, assessments will be conducted before each session.

### Attention Control Arm: House Meetings

Parallel to the intervention arm, participants in clusters randomized to the control arm will complete assessments before content delivery at the first 3 time points. After the first 2 assessments, they will participate in house meetings—group deliberative conversations, sometimes referred to as listening sessions—following a model regularly practiced by TBR [84,85] before participating in the COPE intervention at their third meeting and just after completing their third assessment. The house meeting is a group session that lasts approximately 1.5 hours and engages participants in a discussion about a specific question posed by the facilitators. At the first house meeting, and after administering the first assessment, participants are prompted to discuss “How are you a caregiver in your everyday life? What does it mean to you to be a caregiver?” or a similar question. Each participant is encouraged to share at least once. The facilitator is not necessarily a mental health professional, and no therapy is provided. At the end of the house meeting, participants are reminded of the date for the next meeting. The second house meeting follows the same protocol with a modified prompt question. After completing the third assessment, participants engage in the COPE intervention.

### Study Outcome Measures

We will examine measures of implementation to respond to aim 1 and respond to aim 2 by assessing 3 levels of outcomes (primary, secondary, and tertiary) from the SW-CRT (see Table 1 for a detailed list of outcomes and measures). To address aim 1, we will use the Consolidated Framework of Implementation Research [76] to examine factors that affect intervention implementation and program effectiveness, as well as perceptions of feasibility, acceptability, and cultural appropriateness. We will use standardized measures of acceptability (Acceptability of Intervention Measure [AIM]), appropriateness (Intervention Appropriateness Measure [IAM]), and feasibility (Feasibility of Intervention Measure [FIM]), as well as items related to facilitator preparation and logistics and intervention acceptability developed by the research team. The AIM measures degree of satisfaction with intervention, the IAM its relevance and fit, and the FIM perception of potential for success within an organization. Each construct consists of 5 items rated on a 5-point Likert scale ranging from 1 (completely disagree) to 5 (completely agree). The measures have demonstrated acceptable fit and structural validity and reliability (Cronbach α=0.73-0.88) [86]. We are collecting these data via a survey, interviews, and focus groups conducted with CFs. In addition, we will analyze data from field notes, fidelity observations, interviews, and focus groups with institutional leaders and participants.

**Table 1:** Study outcomes and measures

Measure		Time frame	Type	Source
**Psychological well-being**				
	Depression Anxiety Stress Scale Short Form	In the past week	Primary outcome	Lovibond and Lovibond [87], 1995
	Perceived Stress Scale	In the last month	Primary outcome	Cohen et al [88], 1983
**Social support**				
	Coping Self-Efficacy Scale	—^a^	Secondary outcome	Chesney et al [89], 2006
	Multidimensional Scale of Perceived Social Support	—	Secondary outcome	Zimet, et. al.[90], 1988
**Community connectedness**				
	Communities Advancing Resilience Toolkit	—	Tertiary outcome	Pfefferbaum et al [68], 2020
**Process evaluation**				
	Acceptability of Intervention	—	Acceptability	Weiner et al [86], 2017
	Intervention Appropriateness	—	Appropriateness	Weiner et al [86], 2017
	Feasibility of Intervention	—	Feasibility	Weiner et al [86], 2017

^a^Not applicable.

To address aim 2, we will collect cohort data on the outcome measures at the 5 assessment time points of the SW-CRT: baseline (T1), before session 2 (T2; 1 m after baseline), before session 3 (T3; 2 m after baseline), after the series (T4; 1 month after session 3), and 6 months after the series (T5). Figure 1 provides a visual of the design and timeline of survey measures. As the control arm switches to the COPE intervention after the T3 assessment, assessments pause until 1 month after session 3 so that T4 assessments align (Figure 1). Both arms complete the T5 assessment 6 months after session 3. As part of the CBPR process, the CBPR strategy team deliberated on the outcomes and measurements, coming to consensus on the final survey.

The primary outcomes and their measures assess individual mental health. Perceived stress is assessed with the Perceived Stress Scale (PSS), a 10-item scale that measures the degree to which a person rates situations as stressful [88], for example, how often respondents find their lives “unpredictable,” “uncontrollable,” or feel “overloaded.” Responses are scored on a 5-point Likert scale ranging from 0 (never) to 4 (very often). Total PSS scores range from 0 to 40, with higher scores reflecting higher perceived stress [91]. The 10-item Perceived Stress Scale has high internal reliability (Cronbach α=0.78-0.90) and has been translated and tested in >25 languages [88,91]. Depression, anxiety, and stress are measured with the Depression, Anxiety, and Stress Scale (DASS-21), a set of 3 self-report scales [87]. The 21 DASS-21 items ask about the presence of symptoms over the past week on a scale from 0 (did not apply to me at all) to 3 (applied to me very much or most of the time) [92,93]. Depression scores range from 0 to 4 (normal), 5 to 6 (mild), 7 to 10 (moderate), 11 to 13 (severe), and >14 (extremely severe). Anxiety scores range from 0 to 3 (normal), 4 to 5 (mild), 6 to 7 (moderate), 8 to 9 (severe), and >10 (extremely severe). Stress scores range from 0 to 14 (normal), 15 to 18 (mild), 19 to 25 (moderate), 26 to 33 (severe) and >34 (extremely severe). The DASS-21 has shown high internal reliability (Cronbach α=0.72-0.91) [92-94].

Secondary outcomes and their measures assess coping and social support. Coping self-efficacy is assessed with the Coping Self-Efficacy Scale (CSES), a 12-item measure of confidence in managing stress [89]. The CSES combines 3 subscales measuring distinct dimensions: problem solving, thought stopping, and obtaining social support. Each item is scored on an 11-point scale ranging from 0 (cannot do at all) to 10 (certainly can do). The CSES has high reliability and validity across diverse contexts (Cronbach α=0.76-0.93) [89]. Social support is measured with the Multidimensional Scale of Perceived Social Support (MSPSS), a 12-item measure combining 3 subscales examining perceived support from family, friends, and significant others [90,95]. Each MSPSS item asks at to what degree the respondent agrees that they felt supported by each group and is scored on a 7-point scale ranging from 1 (very strongly disagree) to 7 (very strongly agree). The MSPSS consistently demonstrates internal consistency across diverse populations in the United States and internationally (Cronbach α typically >0.90) [96-98].

The tertiary outcomes measure community resilience and use the Communities Advancing Resilience Toolkit (CART), a 21-item tool that assesses four domains, including (1) connection and caring, (2) community resources, (3) transformative potential, and (4) disaster management [68,99,100]. Each item asks participants to rate their agreement on a 5-point Likert scale from 0 (strongly disagree) to 4 (strongly agree). The 5 domains of the CART have demonstrated validity (connection and caring: Cronbach α=0.83; resources: Cronbach α=0.75; transformative potential: Cronbach α=0.90; and disaster management: Cronbach α=0.91) [101]. Data on relevant covariates, including demographic information (age, race, ethnicity, sex, household income, education, etc), will also be collected at baseline and, in cases where the item may vary over time (eg, self-rated health), repeated at each time point.

### Planned Analyses

#### Aim 1: Evaluate Factors That Affect Intervention Implementation, Efficacy, and Adoption

A mixed methods approach will be used to assess acceptability and feasibility of the COPE intervention. We will survey CFs, using the AIM, IAM, and FIM, to assess acceptability, appropriateness, and feasibility, along with items to assess preparation and satisfaction with the COPE intervention. In addition, intervention fidelity will be monitored in person by research team staff using a standard tool developed for the COPE intervention series. Qualitative assessments will also be conducted throughout the study, especially during the implementation and after the conclusion of every COPE series, and field notes will be taken at workshops. CFs, key leaders, and participants will be interviewed or participate in focus groups using semistructured interview guides.

We will conduct reflexive thematic analysis using the steps outlined and refined by Braun and Clarke [102,103] as a guide to analyze verbatim transcripts or field notes using the MAXQDA qualitative data analysis software. Each of the 6 steps of thematic analysis involves an iterative examination and reexamination of the data to ensure thematic coherence, generating themes from codes and refining the purpose and meaning of themes to clarify their interpretive story [102-104]. This approach permits reflexive discernment of differences across clusters and steps, accounting for nuance given variations in procedures, adjustment to delivery, and development of interpretative stories that convey deeper meaning around collective coping and community resilience [103].

#### Aim 2: Testing of Intervention Effects

Aim 2 will test the effectiveness of the COPE intervention on improvements in mental health symptoms (perceived stress, depression, anxiety, and stress), coping self-efficacy, perceived social support, and community resilience (see Table 1 for measures). The SW-CRT design involves clusters sequentially transitioning from control to intervention, allowing for within-cluster comparisons and accounting for time-dependent effects. The primary analysis for aim 2 will follow an intent-to-treat approach using a linear mixed model to examine within- and between-group differences across time. Missing data will be handled under the assumption that they are missing at random, using restricted maximum likelihood estimation, and we will assess the benefit of multiple imputation across missing values and conduct sensitivity analyses. The linear mixed model will account for the hierarchical structure of the data (eg, individuals, clusters, and time), and include fixed effects for the intervention arm, time, and covariates, such as age, ethnicity, and education. We will also include a random intercept for the clusters to assess between cluster variability and random slopes for time given the variability in time points between the clusters.

At time points 4 and 5, participants in the control arm will have transitioned to the intervention arm. To account for this transition, participants will be categorized based on their current condition (control or intervention) at each time point. This approach ensures that the analysis reflects the condition under which participants were exposed during each survey period. We will also conduct a sensitivity analysis to compare outcomes across time points 1 to 3 (before the switch) to time points 4 to 5 (after the switch). This will allow us to examine intervention effects during the period that the control group is unexposed to the COPE intervention. Results will be presented as regression coefficients with 95% CIs and *P* values, ICCs, and variance estimates for random effects.

### Reflexivity, Transferability, and Generalizability

As a study that uses a CBPR approach, local knowledge is central to optimizing the COPE intervention and study implementation. Our core research team consists of 3 academic researchers, each of whom also have lived experience in Louisiana during a climate disaster, and a CBPR strategy team that brings together individuals with a range of professional and personal backgrounds, including a church pastor and licensed therapist, a retired chemistry professor and civil rights activist, a community organizer, a retired English professor, an executive director of an environmental justice nonprofit, and 2 clinical social workers. The team includes members of different racial or ethnic, gender, and faith backgrounds, each of whom bring lived experience to our collective understanding of mental health challenges in the face of climate disasters and social inequities. Our research team members share identities, experiences, or community ties with our participants, furthering a sense of empathy and responsibility in our approach as our personal experiences are echoed in the stories we hear from friends, family, and neighbors, informing and additionally driving our commitment to this research.

We are approaching our research collaboration from a perspective that recognizes knowledge as accumulated through observation and relationships, reflections, and adaption, which allows us to construct the COPE study and implementation together. Our team believes that community-based mental health support must be integrative, proactive, sustainable, and culturally appropriate. Although we hypothesize that we will find evidence that the COPE intervention effectively improves individual and community mental health outcomes, the trustworthiness of our analyses and interpretation is enhanced by our greater aim, which is to catalyze change that improves mental health and fosters resilience within our community and beyond.

We will ensure confirmability and dependability through extensive intervention monitoring with data collection following structured formats, including fidelity assessment checklist tools and field note guides, as well as interview and focus group guides and transcripts. In addition, we will keep detailed documentation of decisions made throughout the research process and conduct periodic team debriefings. The credibility of our data will be strengthened through our periodic member checking done with monthly meetings of the CBPR team to review the research process and validate findings. Finally, our inclusion of contextual variables and rich description will enhance transferability to allow for application, adaption, or replication.

## Results

The trial testing the COPE intervention funded by the Gulf Research Program of the National Academies of Sciences, Engineering, and Medicine is being conducted from June 2022 to June 2025. The first 9 months of the funding period were dedicated to adapting the RCHC curriculum to develop the COPE intervention model and the pilot. Recruitment was launched with the first cluster of participants in April 2023 and is scheduled to end in spring 2025, with data analysis and peer review submission of results to follow. Our principal focus will be on outcomes related to aim 2, for which we will prioritize individual-level changes on mental health outcomes including depression, anxiety, and perceived stress. Secondarily, we will examine changes in coping, social support, and community resilience. For aim 1, we will examine cluster-level variations and questions regarding COPE implementation.

## Discussion

### Anticipated Findings and Implications

This study aims to evaluate the implementation and effectiveness of COPE, a multilevel, community-based intervention developed to support individuals and communities in mitigating adverse psychological outcomes by enhancing individual coping capacity, social support, and community engagement. There is increasing recognition of the value of multilevel, community-based interventions to address mental health in communities facing compounded effects of inequality and disasters; however, methodologically sound research documenting their implementation and substantiating their effectiveness is limited [69,105]. The study implementing this protocol is among the first to examine the effectiveness and implementation of a multilevel intervention such as COPE in a community that has both experienced prior disasters and is at high risk for future extreme weather events.

Testing the implementation and effectiveness of the COPE intervention holds theoretical and practical significance, contributing to the existing literature on disaster interventions. Multilevel, community-based interventions have become increasingly recognized for addressing the complex interplay between individual, social, and environmental factors that influence disaster resilience and recovery [106-109]. However, few studies examine multilevel interventions, which address the dynamic interplay between individual and systemic factors influencing psychological well-being [110].

At the individual level, COPE focuses on equipping participants with evidence-based skills, such as stress management, emotional regulation, and resilience-building, which can be customized to incorporate local variations on coping practices such as mindfulness or storytelling [111,112]. At the interpersonal level, COPE aims to foster social support through structured group sessions that promote sharing and mutual aid [113]. At the community level, COPE seeks to enhance social capital and engagement by mobilizing local resources and networks, such as partnering with faith-based organizations [114,115]. This approach acknowledges that psychological recovery depends not only on individual factors but also on their concomitant social determinants [69]. At the policy level, COPE aims to inform improvement of existing public health infrastructures to better address community mental health, both in general and after a disaster. In addition, our findings may inform the development of a replicable intervention framework for incorporating facilitator training within local institutions, guiding community health planning; shaping services at the city or state levels; and supporting sustainable, community-driven initiatives throughout the Gulf South region.

Practically, this study will assess how the COPE intervention materials permit tailoring to improve their relevance to discrete groups. Exploring how COPE is adapted at the group-level to include culturally specific symbols, narratives, and traditions, or adjusting focus areas to specific disasters, may further contribute to understanding of the contextualization of disaster mental health intervention approaches [115]. This could provide rich opportunities for future research to not only expand its evidence base and applicability, but also to better understand the interrelationship of cultural processes. Specifically, examining processes of modifying COPE in different contexts to align with diverse cultural norms, values, and beliefs, while maintaining its core principles, could provide insight into how other group-based interventions might better use similar flexible frameworks to expand their cultural relevance [115,116]. Comparative studies examining how COPE is implemented across different cultural and socioeconomic contexts (eg, rural vs urban and collectivist vs individualist communities) could identify best practices for additional contextual tailoring.

Studies are also needed to examine the long-term effects of COPE on individual coping and social capital, including support networks and community resilience. Such studies could also evaluate COPE’s role in enhancing preparedness for future disasters and mitigating long-term mental health challenges. Finally, implementation science studies could explore COPE’s feasibility and scalability in low-resource settings in terms of cost-effectiveness, training requirements, availability of facilitators, and integration with existing community structures [115].

### Potential Limitations

Although this study design presents a rigorous approach to evaluating the effectiveness and implementation of the COPE intervention, limitations should be considered. First, the SW-CRT design may introduce potential biases related to the sequential rollout of the intervention across clusters [74]. Clustering can occur because randomization happens on the group level, potentially generating high intracluster correlation, as participants within the same cluster may respond to COPE more similarly to each other than to those in other clusters. In addition, the clusters are grouped for randomization based on when they could reasonably begin the intervention, then randomized at different time points. This might impact the outcomes due to temporal context particularly as the intervention is introduced sequentially which requires a longer time to completion than would a parallel-group randomized controlled trial [117]. This prolonged duration can increase the risk of external factors, such as in environmental conditions (eg, hurricane season and winter), that may influence the implementation and effectiveness of the intervention [117,118]. We will implement several mechanisms for ensuring fidelity across clusters, including detailed fidelity checklists and monitoring of each facilitator’s initial delivery of the COPE intervention. In addition, to improve generalizability of our findings, we will control for cluster, as well as for step and important seasonal variations.

The prolonged duration of the enrollment and study period can also contribute to attrition and missing data, potentially threatening the robustness and long-term applicability of findings. We will estimate the treatment’s average causal effect to manage missing data effectively [119]. In addition, as is common with intervention studies, long-term impacts may be difficult to assess despite positive short- and medium-term impacts [119,120]. The duration of the study may restrict the ability to assess long-term sustainability and effectiveness of the COPE intervention beyond 6 months. Despite these limitations, the study provides significant insight into the effects of COPE and similar community-based psychoeducational group interventions on mental health in communities at high risk for a disaster, as well as insights into using CBPR in real-world settings to enhance intervention implementation. Together, these findings and insights lay the groundwork for future research on community-based interventions and disaster mental health programs.

### Conclusions

First, this study will provide an understanding of how the multidimensional COPE intervention can address various biopsychosocial (individual, interpersonal, and community) factors that influence well-being, expanding our understanding of the effects of disaster interventions beyond the current focus on individual mental health outcomes [34,121]. Second, the study’s CBPR approach, engaging community members and professionals from the region in close collaboration on all aspects of the study from design to dissemination, increases the cultural relevance, acceptability, and long-term adoption of the COPE intervention, as well as the rigor of the interpretation of its findings. This points to replicable methods for using CBPR to improve upon both the limited tailoring of disaster mental health interventions to specific cultures and contexts, as well as the implementation of intervention research studies. Finally, through its robust examination of the factors that influence implementation of the COPE intervention and its effectiveness in a disaster-prone community in the Gulf Coast, the study will add rich insights into effective delivery strategies for future dissemination or adaptations of COPE and similar multilevel, community-based interventions.

## Appendix

Multimedia Appendix 1 Example facilitator interview guide.

## Abbreviations

AIM: Acceptability of Intervention Measure
CART: Communities Advancing Resilience Toolkit
CBO: community-based organization
CBPR: community-based participatory research
CF: community facilitator
CJoH: Cargivers Journey of Hope
CONSORT-SPI: Consolidated Standards of Reporting Trials–Social and Psychological Intervention
COPE: Communities Organizing for Power through Empathy
CSES: Coping Self-Efficacy Scale
DASS-21: Depression, Anxiety, and Stress Scale
FIM: Feasibility of Intervention Measure
IAM: Intervention Appropriateness Measure
ICC: intraclass correlation coefficient
MHFA: mental health first aid
MSPSS: Multidimensional Scale of Perceived Social Support
PD: psychological debriefing
PFA: psychological first aid
PSS: Perceived Stress Scale
PTSD: posttraumatic stress disorder
PTSS: posttraumatic stress symptoms
RCHC: Resilience and Coping for the Healthcare Community
SW-CRT: stepped-wedge cluster randomized trial
TBR: Together Baton Rouge

## Data Availability

This paper presents the study protocol of a stepped-wedge cluster randomized trial testing the Communities Organizing for Power through Empathy intervention in communities at high risk of experiencing a disaster and does not generate nor analyze an associated dataset, making data sharing nonapplicable. Once the protocol is implemented and data have been collected, the authors will provide access to the detailed protocol and general findings on ClinicalTrials.gov (NCT06093737). Finally, after processing derived data following rigorous procedures to ensure it is deidentified and privacy, rights, and confidentiality of human research participants are protected, deidentified data may be made available upon request.
