# Supplementary material for: The Communities Organizing for Power Through Empathy (COPE) Community-Based Intervention to Improve Adult Mental Health During Disasters and Crises: Protocol for a Stepped-Wedge Cluster Randomized Trial
Source: JMIR Res Protoc. 2025 May 20;14:e63723. doi: 10.2196/63723 (PMC12134696; doi:10.2196/63723)
Supplement: Multimedia Appendix 1 [file resprot_v14i1e63723_app1.docx]

***COPE Community Facilitator Interview Guide***

Thank you for taking the time to discuss your experience as a COPE Community Facilitator with me today. I am going to start the recorder now. **[START RECORDER]** Do you have any questions about your rights as an interviewee? **Do I have your consent to start the interview?**

1. **[Engagement & Interest]** How did you learn about the COPE program?
   - What interested you?
   - How did you learn about the opportunity to facilitate? (if not already shared)
   - What interested you about becoming a COPE Community Facilitator?
2. **[Expectations]** What were your expectations about being a Facilitator prior to facilitating?
   - In terms of time commitment? Payment?
3. **[Curriculum]** How would you describe the COPE curriculum?
   - Which aspects of the curriculum resonated most with the participants?
   - Which aspects of the curriculum resonated least with the participants?
   - If you could rewrite the curriculum, what would you change?
4. **[Training]** Let’s talk about the training you received to facilitate COPE. How did the training prepare you for facilitating COPE in your community?
   - How would you describe your overall feeling of preparedness?
   - After the training, were there any other steps you took to prepare on your own?
   - How often did you communicate with the Program Coordinator (Kelly) after the training and before facilitating? Throughout the process? How about other team members?
5. **[Co-facilitation Preparation]** Please describe how you coordinated with your co-facilitator.
   - Did you meet before facilitating? How often? What did you do in that meeting?
   - What was most helpful to prepare you to facilitate with your co-facilitator?
6. **[Logistics]** How was coordinating the COPE delivery/implementation?
   - Setting dates? Coordinating food? Materials?
   - How often did you communicate with the Project Organizer (Andrea) after the training and before facilitating? Throughout the process? How about other team members?
7. **[Challenges]** Were there any challenges you encountered during delivery of COPE?
   - How did you address them?
8. **[Stories]** Are there any specific experiences that stand out from facilitating the workshop?
   - A story that sticks with you?
   - An incident that occurred?
   - Did you learn anything new about the community?
9. **[mental health]** With regards to mental health, how would you describe the primary issue(s) your group(s) were facing mental health?
   - How would you describe COPE’s role in addressing these challenges?
10. **[S1]** Thinking about Session 1 – the 4-hour workshop focusing on self – is there anything specific to that Session you would add?
    - How prepared did you feel?
    - Which parts of Session 1 resonated most? Least?
11. **[S2]** Thinking about Session 2 – supporting others with the roleplay – is there anything specific to that Session you would add?
    - How prepared did you feel?
    - Which parts of Session 1 resonated most? Least?
12. **[S3]** Thinking about Session 3 – building community support with the asset mapping– is there anything specific about that Session you would add?
    - How prepared did you feel?
    - Which parts of Session 1 resonated most? Least?
    - Did the group make a specific commitment to any actions in that workshop?
    - Have you noticed any changes in your community or organization since?
13. **[community effects]** How has your community responded to COPE?
    - Have you received any feedback from participants that stood out to you?
    - Have you observed any notable changes?
    - Are there any discussions about continuing or expanding the workshop in the future?
    - Have you noticed or heard of any new efforts in your organization? Community?
14. **[barriers]** Were there any barriers/obstacles individuals in your group faced to participating in COPE? How about any barriers with regards to your institution and setting up the COPE series?
    - If so, how did you and the COPE team work to overcome them?
15. **[personal impact]** How has being a facilitator for COPE impacted you personally?
    - Changes in your approach to your own mental health?
    - How have you implemented the curriculum in your personal life?
      1. As a result, what have you noticed?
16. **[changes]** Looking back, is there anything you would have done differently?
17. **[future]** Are there any improvements you suggest for future iterations of COPE?
    - Where would you like to see COPE in the next 5 years?
18. **[access]** What thoughts do you have about how Baton Rouge could improve mental health care?
19. **[advice]** If you could offer advice to future COPE Facilitators, what would it be?
20. **[add]** Is there anything else you would like to add about your experience with COPE?
